# Herbivorous protist growth and grazing rates at in situ and artificially elevated temperatures during an Arctic phytoplankton spring bloom

**DOI:** 10.7717/peerj.5264

**Published:** 2018-07-23

**Authors:** Susanne Menden-Deuer, Caitlyn Lawrence, Gayantonia Franzè

**Affiliations:** Graduate School of Oceanography, University of Rhode Island, Rhode Island, United States of America

**Keywords:** Arctic ecosystem, Food-web dynamics, Spring bloom, Grazing, Temperature response, Plankton production, Arctic warming, Heterotrophic protists

## Abstract

To assess protistan grazing impact and temperature sensitivity on plankton population dynamics, we measured bulk and species-specific phytoplankton growth and herbivorous protist grazing rates in Disko Bay, West Greenland in April-May 2011. Rate estimates were made at three different temperatures in situ (0 °C), +3 °C and +6 °C over ambient. In situ Chlorophyll *a* (Chl *a*) doubled during the observation period to ∼12  µg Chl *a* L^−1^, with 60–97% of Chl *a* in the >20 µm size-fraction dominated by the diatom genus *Chaetoceros.* Herbivorous dinoflagellates comprised 60–80% of microplankton grazer biomass. At in situ temperatures, phytoplankton growth or grazing by herbivorous predators <200 µm was not measurable until 11 days after observations commenced. Thereafter, phytoplankton growth was on average 0.25 d^−1^. Phytoplankton mortality due to herbivorous grazing was only measured on three occasions but the magnitude was substantial, up to 0.58 d^−1^. Grazing of this magnitude removed ∼100% of primary production. In short-term temperature-shift incubation experiments, phytoplankton growth rate increased significantly (20%) at elevated temperatures. In contrast, herbivorous protist grazing and species-specific growth rates decreased significantly (50%) at +6 °C. This differential response in phytoplankton and herbivores to temperature increases resulted in a decrease of primary production removed with increasing temperature. *Phaeocystis* spp. abundance was negatively correlated with bulk grazing rate. Growth and grazing rates were variable but showed no evidence of an inherent, low temperature limitation. Herbivorous protist growth rates in this study and in a literature review were comparable to rates from temperate waters. Thus, an inherent physiological inhibition of protistan growth or grazing rates in polar waters is not supported by the data. The large variability between lack of grazing and high rates of primary production removal observed here and confirmed in the literature for polar waters implies larger amplitude fluctuations in phytoplankton biomass than slower, steady grazing losses of primary production.

## Introduction

Herbivorous protists are key grazers in marine microbial food webs. While phytoplankton generate approximately 50% of the total, global primary production (Field et al., 1998), grazing by herbivorous protists removes on average 2/3 and constitutes the single largest loss factor of marine primary production (Steinberg & Landry, 2017). Research to date suggests that planktonic food webs in the Arctic are as complex and important to ecosystem function as elsewhere in the global ocean (Garrison & Buck, 1989; Garrison, Buck & Gowing, 1993; Nielsen & Hansen, 1995; Rivkin, Anderson & Lajzerowicz, 1996; Sherr, Sherr & Fessenden, 1997; Edwards, Burkill & Sleigh, 1998). However, because high latitude regions are less accessible and working conditions are often more difficult, very few data on plankton population dynamics and empirical measurements of protistan growth and grazing rates exist for polar regions. Thus, estimates of Arctic food web dynamics and rates of biogeochemical cycling of major elements are poorly constrained.

Assessment of the relative rates of plankton growth and grazer induced mortality is key to understanding high latitude plankton population dynamics. Long standing hypotheses suggest that the low temperatures reduce bacterial remineralization of organic matter and yield higher secondary production rates than temperate waters (e.g., Pomeroy & Deibel, 1986). Rapid, anthropogenic increases in atmospheric carbon dioxide concentrations have caused a series of dramatic changes in global environmental conditions, with significant effects on the functioning of ocean ecosystems (Hoegh-Guldberg & Bruno, 2010). Temperatures in the Arctic have increased at approximately twice the global average rate (Hansen et al., 2006), and sea ice cover has decreased rapidly (Nghiem et al., 2007), leading to rapid changes in the timing and duration of light and nutrient availability to Arctic plankton. As elsewhere in the ocean, the carbonate system in the Arctic is changing, due to absorption of atmospheric CO_2_ (e.g., Aberle et al., 2013). Melting sea ice will change the salinity of surface waters as well as water column stratification. These changes in environmental conditions likely alter plankton species composition and abundance patterns. Significant increases in temperature have also been recorded in Disko Bay (Hansen et al., 2012), the site of this study. Arctic temperature increases are in line with the ‘Arctic amplification’ highlighted in the IPCC 4th Assessment Report (AR4) (IPCC, 2013). Magnification of global warming trends in high latitudes hold potentially serious consequences for Arctic planktonic food webs, including shifts in primary production and the transfer of matter and energy to higher trophic levels or towards export production. Assessing the long term ramifications of these changes is challenging. However, as the frequency and the intensity of environmental disturbances and fluctuations are increasing it becomes pivotal to investigate the short-term response of microbial communities to rapid changes in environmental conditions, including temperature, and subsequent effects on biogeochemical fluxes.

Based both on few direct, empirical measurements and derived estimates of grazing rates, it appears that grazing impact by herbivorous protists in polar regions is often lower than the global average of ∼67% (e.g., Levinsen, Nielsen & Hansen, 1999; Caron et al., 2000; Sherr & Sherr, 2009; Calbet et al., 2011b). It is noteworthy, however, that measurements of high-latitude grazing rates are often highly variable. In a study by Sherr, Sherr & Hartz (2009), the average grazing impact was indeed low, only ∼20% of primary production was consumed. However the distribution of grazing rates was not uni-modal, many low rate estimates were punctuated by few, very high rates. The largest grazing rates removed up to 120% of daily phytoplankton primary production consumed (Sherr, Sherr & Hartz, 2009). Substantial herbivory was also reported in the Barents Sea where herbivorous protists removed >100% of phytoplankton production in North Atlantic-influenced water, while the impact in Arctic water was lower but still considerable (57%) (Franzè & Lavrentyev, 2017). Further measurements of near maximal herbivorous protist grazing rates at low temperatures (<2 °C), have been reported also in studies from diverse environments or wintertime conditions (Putland, 2000; Lawrence & Menden-Deuer, 2012; Sherr, Sherr & Ross, 2013; Morison & Menden-Deuer, 2017), suggesting that herbivorous protists can remove substantial fractions of primary production, even at low ambient temperatures. These empirical observations demonstrate that high grazing rates by herbivorous protist are physiologically possible, even under ice.

Few attempts have been made to measure herbivorous protist growth rates at high latitudes due to both methodological and technical constraints. However, the largest study to-date of species-specific, herbivorous protist growth rates along a temperature gradient in the Barents Sea documented that maximum growth rates were observed at temperatures <5 °C (Franzè & Lavrentyev, 2014). In a Bering Sea study, herbivorous growth rates even exceeded phytoplankton growth, 0.43 d^−1^ vs. 0.22 d^−1^, respectively (Sherr, Sherr & Ross, 2013). Comparably high herbivorous growth rates at low temperatures have been measured in the Southern Ocean (Bjornsen & Kuparinen, 1991).

Despite the generally sparse empirical data, enough evidence exists to conclude that low temperatures do not consistently depress herbivorous protist growth, or as documented in several studies cited above, grazing rates. Complex interactions between biotic and abiotic factors, such as community composition and abundance, type and quantity of suitable prey, temperature and nutrient availability may affect the measured physiological rates (Menden-Deuer & Kiørboe, 2016). For example, Chen et al. (2012) identified significant co-variation between effects of ambient Chlorophyll *a* (Chl *a*) concentration and temperature on the magnitude of grazing pressure and Poulin & Franks (2010) recognized many biotic and abiotic factors as important parameters in deciphering grazing interactions.

To elucidate effects of temperature and rapid temperature changes on plankton population dynamics and primary production in Arctic waters, we measured herbivorous protist growth and grazing rates and their impact on phytoplankton production at ambient, freezing temperatures and in response to an abrupt increase in temperature by subjecting incubation experiments to a shock warming treatment. Plankton can be subject to abrupt changes in both chemical and physical conditions, such as temperature and nutrient availability, as result of the increased occurrence of extreme events, which can rapidly alter, for example, water column stratification and fresh water input. Thus, measurements of un-acclimated rates could provide useful information about the effect of short-term environmental changes on phototrophic and herbivorous organisms. In April–May 2011, over the course of an intensifying spring bloom development in Disko Bay, Western Greenland, we measured phytoplankton growth and herbivorous protist growth and grazing rates at in situ temperatures. In addition, growth and grazing rates were measured in un-acclimated, short-term incubations at temperatures of +3 °C and +6 °C. While phytoplankton showed the expected increase in growth rate with increasing temperature (Eppley, 1972) maximum herbivorous growth and grazing rates decreased at increasing temperature, suggesting differences in acclimation capacity of the photo- and heterotrophs to short-term shock responses. The implications for food web dynamics are discussed. Few observations of high rates of herbivorous growth and grazing at freezing temperatures provided further support for the importance of protistan predators in Arctic microbial food webs and suggest that low temperatures do not universally inhibit herbivorous grazing, because of inherent physiological constraints.

## Materials and Methods

### Water sampling

**Figure 1:**
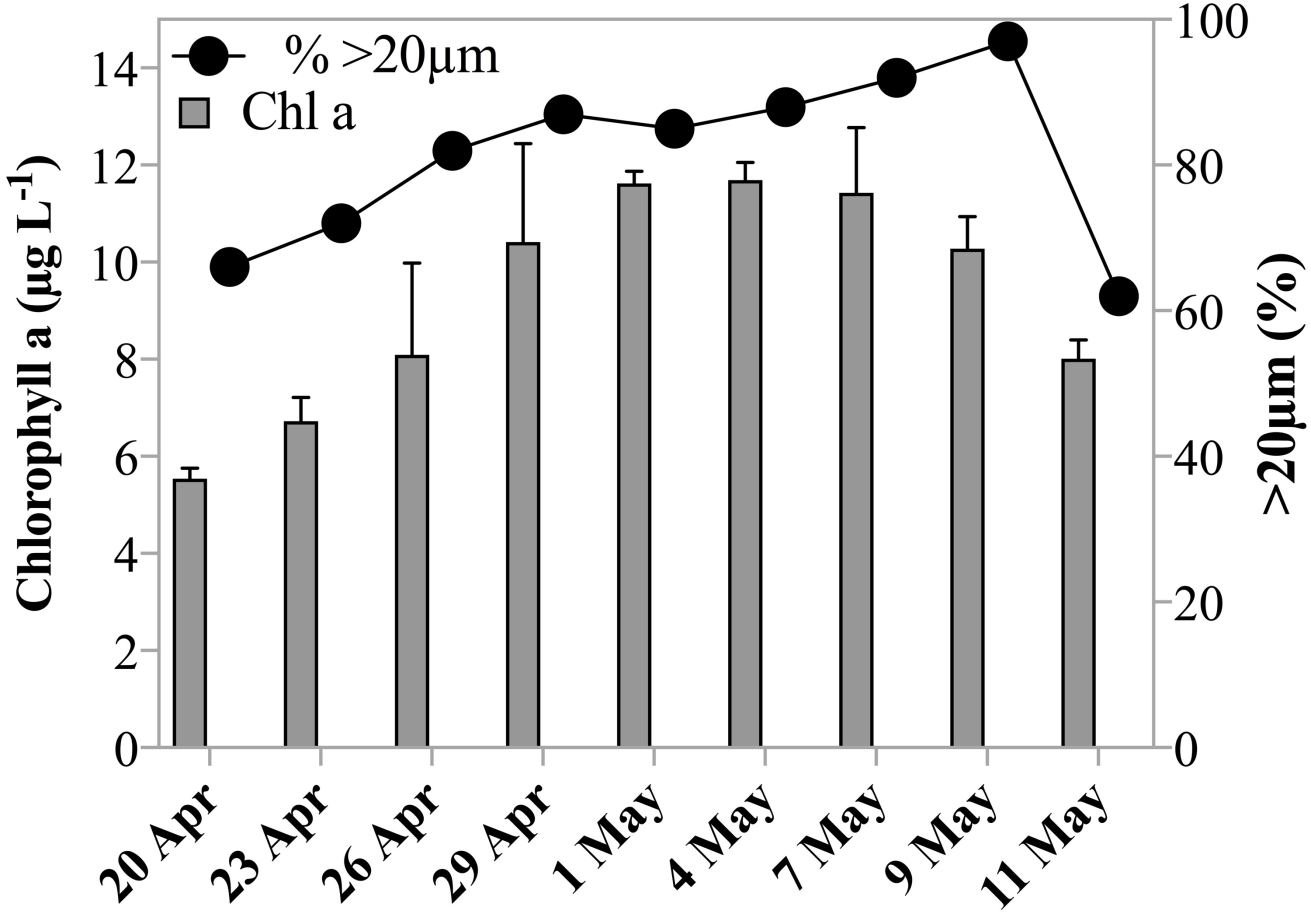
Chl a concentration and size fraction. Chl a concentration (µgL^−1^) within the subsurface fluorescence maximum during the sampling period. Error bars are one standard deviation of the mean of triplicate extracts. Black circles indicate fraction of Chl a measured in the >20 µm size fraction.

Between April 20th and May 11th 2011, 27 dilution experiments were conducted at a 250 m deep coastal site, 1 nautical mile south of Disko Island, Western Greenland (N 69°11, W 53°14); (see Fig. 1 in Levinsen, Nielsen & Hansen, 2000). Source water was collected every 2–3 days. Daily changes in ice cover required up to 3 km changes in sampling location. Water column profiles of temperature, salinity, in situ PAR and Chl *a* fluorescence were acquired with a SBE19plus CTD. Water samples were collected with Niskin bottles from the fluorescence maximum at depths ranging between 15 and 40 m (Table 1), transferred into to 20 L polycarbonate carboys using submerged silicone tubing and stored in the dark for transport to the laboratory. Water samples froze partially during transport to the laboratory on the first three sampling dates; samples on the first date froze completely and had to be thawed. Remarkably, actively swimming protists were observed in the melt water thus, we chose to conduct experiments with this source water. However, freezing could have negatively impacted the plankton community in terms of abundance, composition and physiology on these three occasions.

**Table 1:** Environmental and biological conditions in Disko Bay, Greenland during the campaign in April–May 2011. Phytoplankton growth (µ) and herbivorous grazing (g) rates (d^−1^) were measured at in situ temperatures and at +3 °C and +6 °C treatments.

Date	Depth m	Temp. °C	Chl a µg L^−1^	In situ °C			+3°C			+6°C	
				µ	g		µ	g		µ	g
21 Apr	25	−1.7	5.54	−0.27	0.00		−0.09	0.11		−0.07	0.27
23 Apr	25	–	6.73	0.36	0.35		0.11	0.26		0.06	0.00
26 Apr	15	−1.5	8.09	0.06	0.51^*^		0.04	0.47^*^		−0.16	0.09^*^
29 Apr	15	−1.4	10.42	−0.02	0.00		0.09	0.00		0.07	0.00
1 May	15	−1.5	11.63	0.07	0.10		0.03	0.00		0.07	0.00
4 May	25	−1.4	11.69	0.62^*^	0.58^*^		0.64^*^	0.67^*^		0.65^*^	0.52^*^
7 May	25	–	11.43	0.14^*^	0.07		0.20^*^	0.33		0.20^*^	0.15
9 May	–	–	10.28	0.18^*^	0.00		0.26^*^	0.03		0.17^*^	0.00
11 May	–	−0.9	8.02	0.07^*^	0.11^*^		0.20^*^	0.20^*^		0.19^*^	0.07^*^

**Notes.**

Asterisks denote rates significantly different from zero.

### Plankton population dynamics

Phytoplankton growth rates and herbivorous grazer-induced mortality rates were measured using the dilution method (Landry & Hassett, 1982) in a two-point modification using whole seawater (WSW) and a diluted fraction containing 10% WSW (Worden & Binder, 2003; Landry et al., 2008; Strom & Fredrickson, 2008; Lawrence & Menden-Deuer, 2012). The validity of this abbreviated approach in providing statistically indistinguishable growth and grazing estimates from the multi-point dilution approach has been demonstrated for both linear and non-linear feeding responses (Strom & Fredrickson, 2008; Chen, 2015; Morison & Menden-Deuer, 2017).

In total there were 9 dates on which dilution experiments were performed. For each of those dates, experiments were run at 3 experimental temperatures (see below). At the lowest temperature, incubations were performed with and without added nutrients to test for nutrient limitation. To generate the WSW fraction, source water was screened through a 200 µm Nitex mesh to remove macro-zooplanktonic predators. Subsequently WSW (20 L) was gravity filtered through a 0.2 µm filter cartridge to generate diluent for the 10% WSW dilution. Both dilutions were prepared in single carboys to minimize variation among replicates and then gently siphoned into 1.8 L polycarbonate incubation bottles. Both dilutions were incubated in parallel with and without added nitrate (5 µM) and phosphate (0.5 µM) to account for potential nutrient limitation on rate estimates. All bottles and silicon tubing used were acid washed, rinsed with deionized water and then filtered seawater to eliminate toxicity effects on protists (Price et al., 1986).

Samples were processed as quickly as possible and added to incubators within 1–3 h after returning from the cruise. Triplicate 1.8L bottles of both WSW and 10% WSW were incubated for 24 h in laboratory vans under cool fluorescence light with a 20:4 light-dark cycle. All bottles were placed inside neutral density mesh screen bags to simulate the light level at sampling depth (10–15 µmol photons m^−2^ s^−1^) and were manually rotated every 4 h.

Chl *a* was extracted from triplicate subsamples collected when bottles were filled initially (T_0_) and in triplicate from each of the triplicate dilution bottles after 24 h (T_F_). In addition, the size structure of the initial phytoplankton community was characterized from triplicate size-fractionated Chl *a* samples (>0.7 GF/F and >20 µm). The extraction method followed Graff & Rynearson (2011) with the exception of the use of 95% ethanol as a solvent (Jespersen & Christoffersen, 1987). The volume filtered ranged from 50 to 200 mL depending on phytoplankton abundance and dilution.

For microscopy analysis of species composition and biomass via size, 100 mL of WSW from both T_0_ and T_F_ was preserved with 2% acid Lugol’s iodine (final concentration; Menden-Deuer, Lessard & Satterberg, 2001). Counts of dominant phytoplankton >5 µm in diameter were made with a 1 mL Sedgwick-Rafter slide, a minimum of 300 cells per sample were counted. Less abundant species, and herbivorous protists were counted in 50 mL Lugol’s fixed samples settled following the Utermöhl method. The entire surface of the chamber was scanned under an inverted microscope at 100–200×  to ensure adequate sample size. However when low cell numbers were encountered, multiple species were binned to increase the confidence of the rate estimate. Taxonomic identification was based on morphological characteristics (Dodge, 1985; Tomas, 1997; Lee, Leedale & Bradbury, 2000; Horner, 2002). Protist biovolume was calculated from linear dimensions by approximating geometric shapes and converted to carbon using the conversion equations in Menden-Deuer & Lessard (2000). Microscope counts were made for samples from experiments conducted between April 23rd and May 7th. No counts were made for experiments on April 20th, or May 7th and 11th.

Data treatment for the dilution experiment based rate estimates follow procedures outlined in Morison & Menden-Deuer (2017). In brief, phytoplankton growth rates measured in the 10% WSW were considered to be a reasonable estimate for the instantaneous growth rates unaffected by grazing. Thus, phytoplankton growth rate (µ, d^−1^) was calculated as *μ* = 1∕*t*∗ln(*C*_t_∕*C*_0_), with C_t_ and C_0_ the final and initial Chl *a* concentration respectively and t the time elapsed in days. Herbivorous grazing rate (g, d^−1^) was calculated as the difference between µ measured in the highly diluted (µ10%) and WSW (*μ*_WSW_) fractions *g* = *μ*_10%_ − *μ*_WSW_. For cases where no grazing was measured, as indicated by no difference in µ in the two dilutions or greater growth in the WSW fraction, g was recorded as zero and µ averaged across dilution levels. Statistical analyses were conducted to ensure conclusions were not altered irrespective of whether grazing rates were set to zero or unaltered. Averages and plots are based on the data treatment where lack of grazing is recorded as *g* = 0.

Phytoplankton growth rates from nutrient amended and non-amended experiments were compared in a paired *t*-test to determine whether nutrient limitation occurred. When nutrient limitation was present, grazing rates were determined from the nutrient amended treatment and phytoplankton growth rates from the non-amended treatment following the methods outlined in Landry et al. (1998). Otherwise, rates from both amended and non-amended treatments were combined. Species-specific growth rates for phytoplankton and herbivorous protists were calculated as the ratio of natural log transformed cell abundances over time.

The ratio between grazing rate (g, d^−1^) and phytoplankton growth rate (*μ*, d^−1^) was used to estimate percent primary production consumed (% PP = g/µ*100). Dates on which no phytoplankton growth or herbivorous grazing was measured were not used to calculate %PP consumed.

### Temperature treatments

Samples were incubated at three temperature treatments: in situ (0 °C), +3 °C and +6 °C over ambient. Water temperature for the three treatments was maintained as follows: the in situ treatment temperature was maintained through addition of snow to the incubation basin and was 0 °C (±0.0 °C). The +3 °C treatment was left to equilibrate with the ambient walk-in incubator air temperature and was 3.9 °C on average (±0.2 °C), and the +6 °C treatment temperature was maintained by a flow through water bath and was 6.0 °C on average (±0.2 °C).

Incubation bottles were not acclimated to prior temperature. Thus, the transfer from in situ to incubation temperature could have induced a temperature dependent shock-response in the plankton communities and affected the rates measured. The magnitude of this shock would have been highest in the highest temperature treatment, because the shift in temperature was greatest relative to in situ. Although acclimation could have reduced the potential shock induced by the target temperature, delay in commencement of the experiments would have extended the incubation duration and thus could have altered the species composition and nutrient concentrations. The experimental results from the temperature manipulation treatments should be viewed cautiously in light of the impossibility of acclimating whole communities to rapid changes in environmental conditions in a manner that preserves the integrity of the sampled community and minimizes incubation effects, including nutrient limitation and lack of immigration/emigration (see discussion and Grear et al., 2017). These limitations on experimental protocol are common and affect other methods that by necessity isolate a water sample, such as mesocosms.

### Statistical analysis

Temperature effects on phytoplankton growth and herbivorous grazing rates were examined using 2-way ANOVA, with incubation temperature and date as factors. Temperature effects on taxon-specific growth rates for a subset of dates were analyzed using a 2-way ANOVA with taxa and temperature as factors. Post-hoc analyses used the Bonferroni approach and examined interaction effects, when significant. Regression analysis used a linear, type-II model (i.e., both variables measured with error). Effects of nutrient addition on phytoplankton growth rates were determined with a paired *t*-test. Normality of data distributions was assessed with a Lilliefors test. All analyses were assigned statistical significance at *p* < 0.05.

## Results

### Environmental conditions

Mid-day air temperature increased continuously over the study period from −20 °C to +5 °C. Although no quantitative measurements were made, sea ice covered approximately 50% of Disko Bay in mid-to late April and ice cover decreased steadily until conditions were nearly ice free in mid May. However, water column conditions were surprisingly constant despite the observed changes in atmospheric conditions, tidal exchange and exact sampling location: water temperature consistently remained below −1 °C at the surface while steadily increasing with depth and reaching ∼1 °C at 150 m. Surface salinity remained between 33 and 33.5 and was nearly unchanged between late April and mid-May. Salinity increased with depth up to 34.5 at 150 m. Maximum surface irradiance was 200 µmol photons m^−2^ s^−1^ during late April and increased to 400 µmol photons m^−2^ s^−1^ in May. Irradiance was 10–15 µmol photons m^−2^ s^−1^ at sampling depths. A pronounced peak in Chl *a* induced fluorescence was observed on all sampling days. The mean depth of the peak signal varied between 15 and 40 m and the vertical extent was typically >15 m.

### Plankton biomass and composition

Chl *a* concentrations during the sampling period indicated that the experiments were conducted over the course of a developing spring bloom, i.e., increasing phytoplankton biomass. On the first day of sampling Chl *a* had already reached 5.5 µg L^−1^, by May 1st 2011 the concentration increased to 11.6 µg L^−1^ and decreased slowly thereafter to 8.0 µg L^−1^ on the last day of sampling (Table 1, Fig. 1). Phytoplankton >20 µm contributed initially 66% to the Chl *a* concentration. The fraction of microplankton in the Chl *a* concentration reached a maximum of 97% on May 9th 2011 (Fig. 1), despite the breakage of large *Phaeocystis* colonies during filtration, and thus a potential underestimate of the large size fraction. Phytoplankton were largely composed of diatoms, prymnesiophytes and silicoflagellates and, species composition was similar over the investigation period.

**Figure 2:**
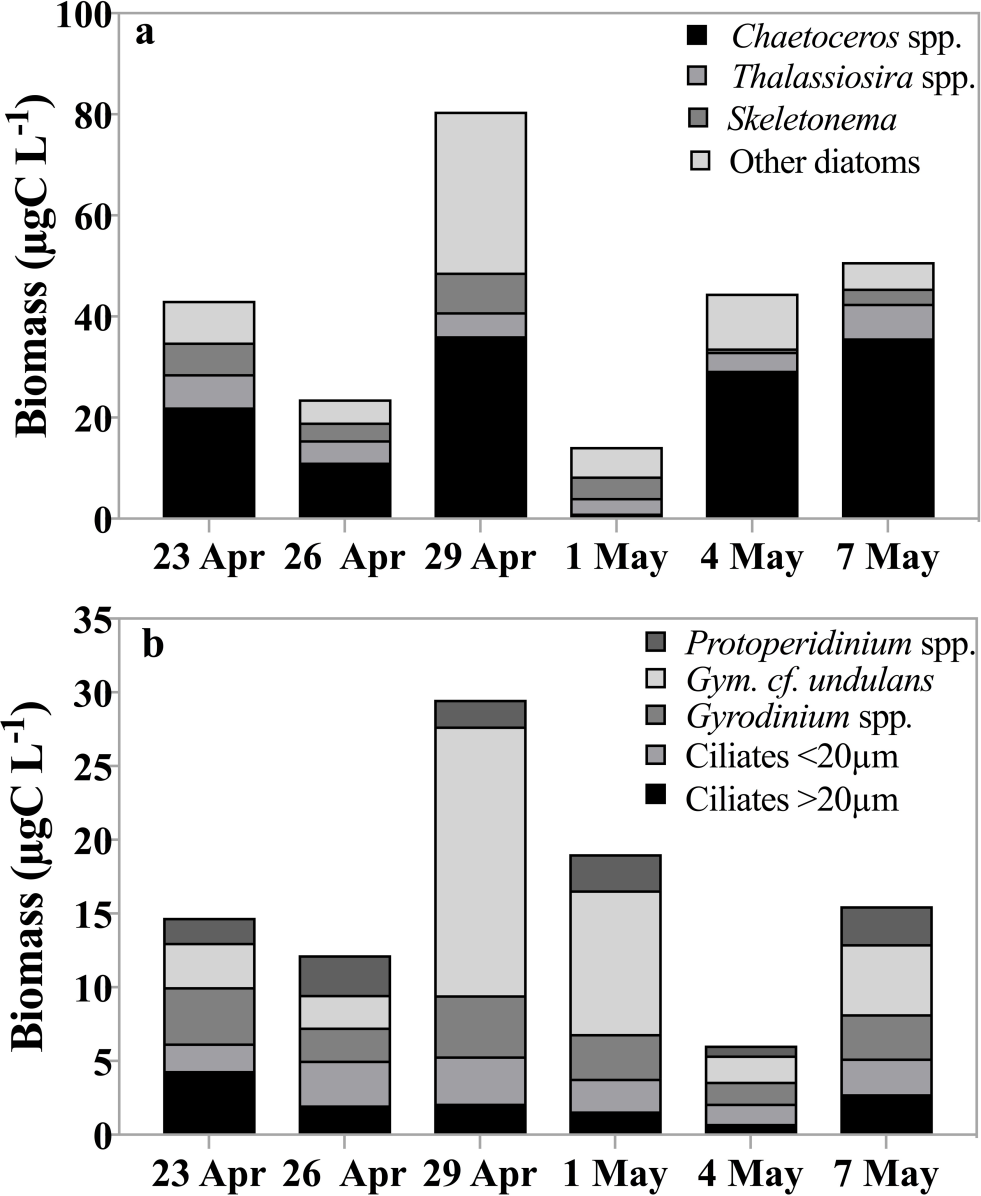
Carbon biomass of dominant taxa. Carbon biomass of the dominant (A) phytoplankton and (B) herbivorous protist taxa (µg C L^−1^) over time (date). Source samples were derived from the fluorescence maximum.

Biomass of the abundant, not total, phytoplankton taxa ranged between 24 µg C L^−1^ and 80 µg C L^−1^ and was dominated by three diatom genera, *Chaetoceros* spp., *Thalassiosira* spp. and *Skeletonema* spp. (Fig. 2A). This estimate does not reflect the total phytoplankton biomass, which, assuming a C:Chl *a* ratio of 30 (Sherr et al., 2003), could have reached up to 350 µg C L^−1^. *Phaeocystis* abundance ranged between 38 to 104 colonies per ml, with a minimum observed on May 4th and the maximum on April 29th.

Biomass of the common herbivorous protist (including both herbivorous and mixotrophic taxa) at the fluorescence maximum averaged 16 µg C L^−1^, ranging 4-fold over the observation period, from 6 µg C L^−1^ to 30 µg C L^−1^ on May 4th and on April 29th respectively (Fig. 2B). Dinoflagellates, especially athecate species including *Gyrodinium* and *Gymnodinium* spp. contributed between 60–80% to the total biomass, whereas ciliates contributed 20–40%. When biomass peaked, dinoflagellates exceeded ciliate biomass by ∼30%. Within the dinoflagellates, *Gymnodinium* spp. contributed up to 62% (18 µg C L^−1^) to total biomass while *Protoperidinium* spp. and *Gyrodinium* cf. *undulans* represented between 6 and 22% and 14 and 26% of total biomass, respectively. Oligotrich ciliates smaller than 20 µm contributed between 11 and 25% of the total biomass, whereas larger ciliates (>20 µm), including both aloricate and loricate species, varied between 1 µg C L^−1^ and 4 µg C L^−1^, or between 7 and 29% of total biomass. Both ciliate groups contributed on average 15% to total herbivorous protist biomass. It is worth noting that during our study no diatom-feeding amoebae or parasitic flagellates that could have contributed to grazing pressure were observed (Sherr, Sherr & Ross, 2013).

### Plankton population dynamics

**Figure 3:**
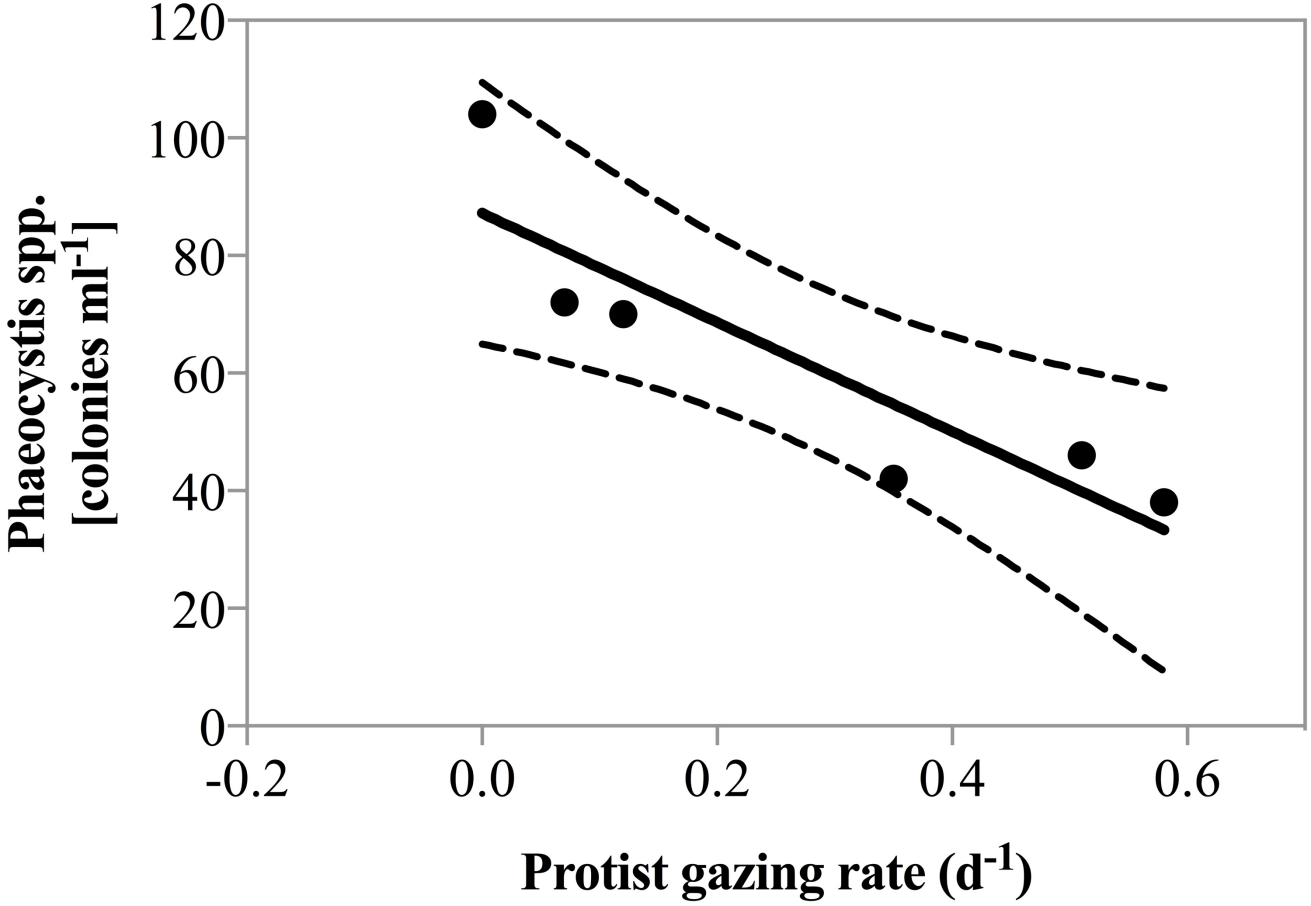
Grazing rate as a function of Phaeocystis spp. colony abundance. Regression of total grazing rate (*d*^−1^) vs. abundance of *Phaeocystis* spp. colonies. There was a significant negative correlation between the abundance of *Phaeocystis* colonies and measured grazing rate (*y* = 92.8∗*x* + 87.2, *p* = 0.015, *r*^2^ = 0.81).

Net phytoplankton growth rates ranged between −0.27 ± 0.25 and 0.62 ± 0.05 d^−1^. During all dates in April, there was no enhancement in phytoplankton growth rate due to nutrient addition. Beginning May 1st phytoplankton growth was significantly faster in nutrient amended treatments relative to the un-amendment control (0.25 vs. 0.42 d^−1^; paired *t*-test, *p* = 0.01). Grazing rate varied considerably ranging from 0, measured on several dates, to 0.58 ± 0.08 d^−1^, measured on May 4th (Table 1). Predator biomass, or changes therein could not explain the magnitude of grazing rates measured. As a matter of fact, herbivorous protist biomass was lowest when grazing pressure peaked on May 4th 2011 and a significant negative correlation between predator biomass and predation pressure was observed (*r*^2^ = 0.74, *p* = 0.03). However, it is noteworthy that on May 4th the highest contribution of the potential cytotoxic genera *Phaeocystis* and *Skeletonema* was measured and a negative correlation between grazing and the number of *Phaeocystis* colonies was also detected (Fig. 3, *r*^2^ = 0.81, *p* = 0.015).

The fraction of primary production removed by grazing was highly variable. Herbivorous grazing rates did not exceed phytoplankton growth rates on most dates. However, when grazing was measurable on April 26th, May 4th and May 11th (Table 1), between 94 and >100% of daily primary production was consumed by herbivorous protists. Percent primary production consumed exceeding 100% were observed only when inherent phytoplankton growth rates were low. For the remainder of the days, the low impact of herbivorous protist grazing on primary production is well supported by the observation of in situ accumulation of phytoplankton biomass.

### Temperature effects

**Figure 4:**
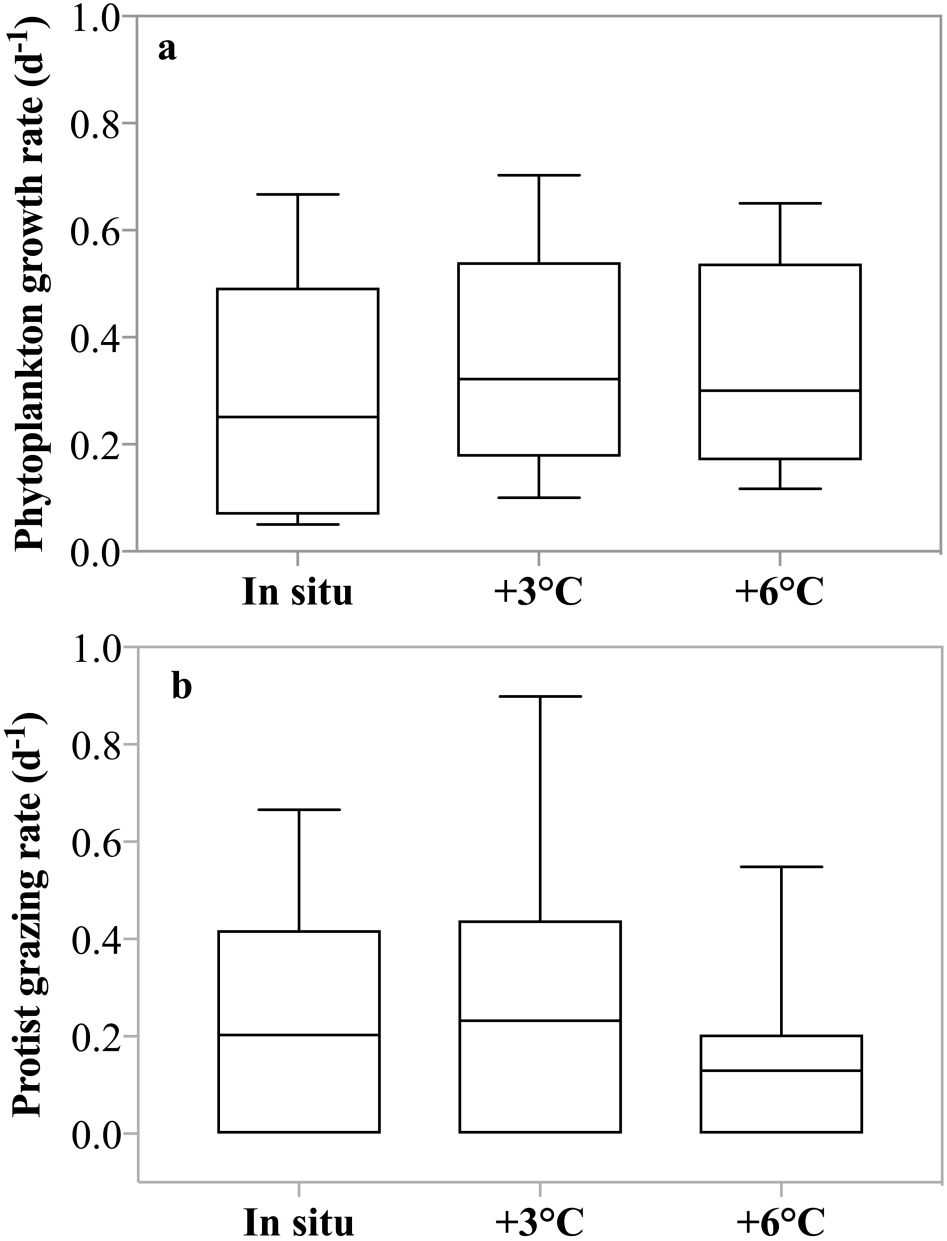
Temperature dependence of phytoplankton growth and herbivorous grazing rates. (A) Phytoplankton growth rates (d^−1^) in the 3 temperature treatments: in situ, +3 °C and +6 °C. Data shown are all replicates from all experiments with positive growth rates, after May 1st. (B) Protist grazing rates (d^−1^) for all incubations at in situ, +3 °C and +6 °C. Boxes indicate the mean and upper and lower 25th and 75th percentiles and the whiskers contain 95% of the data range.

No significant difference in net phytoplankton growth rates among incubation temperatures was observed over the entire sampling period (2-way ANOVA, *p* = 0.81); average growth rates were *μ* = 0.13, 0.16, and 0.13 d^−1^ (±0.13 d^−1^) at in situ, +3 °C, and +6 °C, respectively. When only experiments where phytoplankton growth was greater than zero at all three temperatures (May 4th to 11th) were included in the analysis, a significant enhancement (2-way ANOVA, *p* < 0.001) of growth at both +3 °C and +6 °C relative to the in situ temperature was observed (Fig. 4A). There was no interaction between dates and temperature. During this latter period, phytoplankton growth rates increased by ∼20%, *μ* = 0.25, 0.32, and 0.30 d^−1^ at in situ, +3 °C, and +6 °C respectively, even though nutrient limitation was likely suppressing phytoplankton growth.

Herbivorous grazing rates were significantly reduced at +6 °C compared to the two lower incubation temperatures, with *g* = 0.20, 0.23, 0.13 d^−1^ at in situ, +3 °C, and +6 °C respectively (*p* = 0.03, 2-way ANOVA, Fig. 4B). There was no interaction between dates and temperature. This suppression was even more pronounced, when only experiments with detectable grazing impact at all treatment temperatures were included (Apr 26th, May 4th, and 11th) with *g* = 0.40, 0.45, 0.23 d^−1^ at in situ, +3 °C, and +6 °C, respectively (*p* = 0.01, 2-way ANOVA). It is noteworthy that average grazing rate at +6 °C was 50% lower than grazing rate measured at the 2 lower temperature treatments.

Analysis of the *potential* impact of the un-acclimated, short-term temperature effects on plankton population dynamics revealed an interesting shift in the fate of primary production as a function of temperature. The overall fraction of primary production removed decreased with increasing temperature. This decrease in primary production removed is due to the asynchronous response of increased phytoplankton growth with increasing temperature paired with decreasing herbivorous grazing with increasing temperature. For the data from May 4th, during the peak of the phytoplankton bloom and when maximum grazing rates were measured at all three temperature treatments ∼100% of primary production was removed at in situ temperature, whereas only 75% was removed at +6 °C.

**Figure 5:**
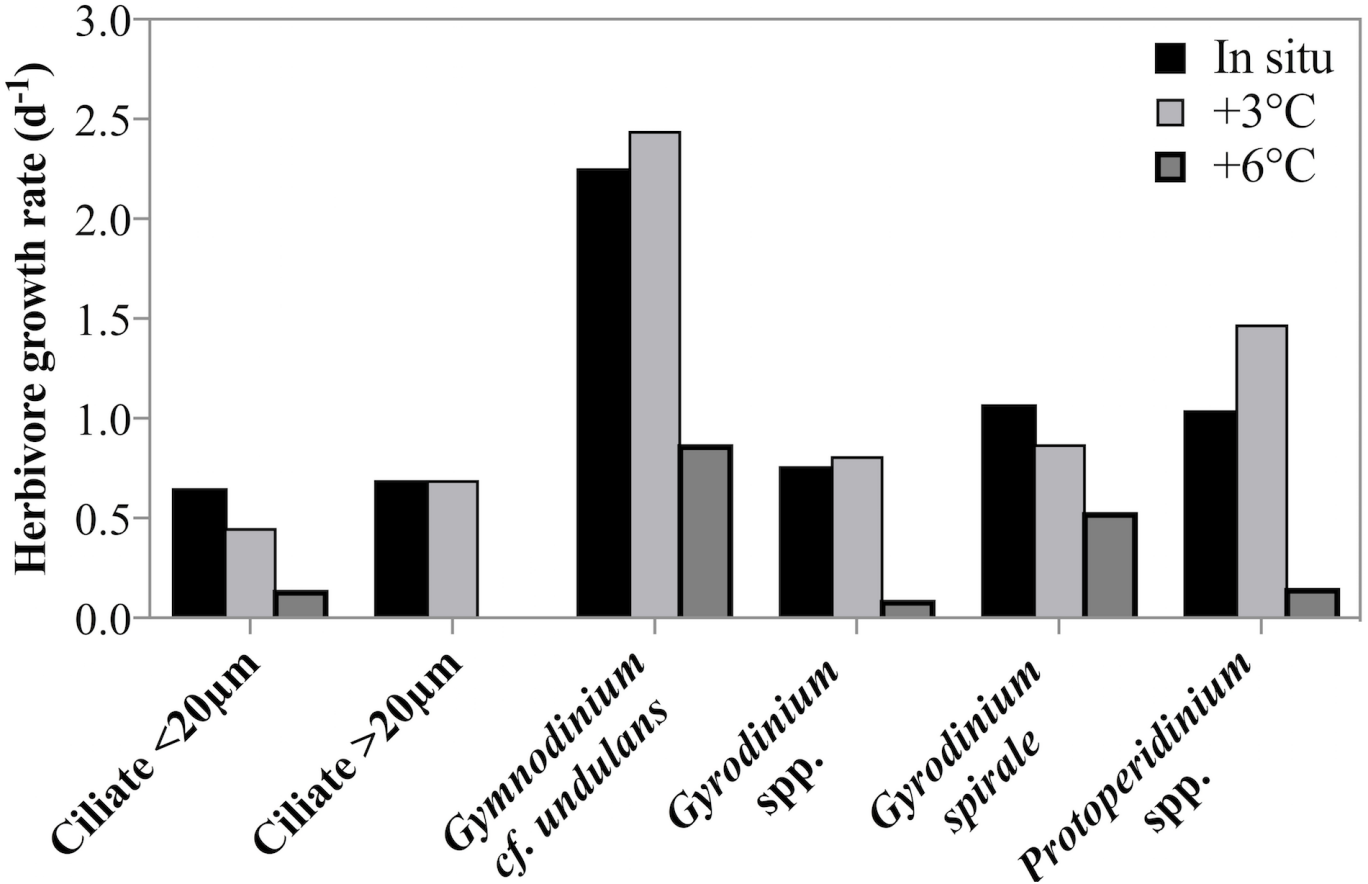
Herbivorous protist growth rates across 3 treatment temperatures. Growth rates (d^−1^) of six groups of herbivorous protists at 3 treatment temperatures on May 4th 2011 when the highest grazing rates were measured. For all groups there was a significant decrease in growth rate between in situ and +6 °C. For ciliates <20 µm and *Gyrodinium spirale* the decrease in growth rate was significant also at +3 °C.

Estimates of taxon-specific growth rates revealed that all of the five herbivorous protist species or groups grew significantly faster at lower temperatures (2-way ANOVA, *p* = 0.001, Fig. 5). It is also noteworthy that both ciliates and dinoflagellates achieved very high growth rates despite the low temperature and many taxa or groups exceeded a doubling a day. Ciliate growth, although considerable, was lower than dinoflagellate growth rate. At in situ temperatures cross-taxon averages were *μ* = 0.44 d^−1^ and *μ* = 0.75 d^−1^ for ciliates and dinoflagellates respectively, excluding the very fast growing *G. cf undulans* from the group average.

**Figure 6:**
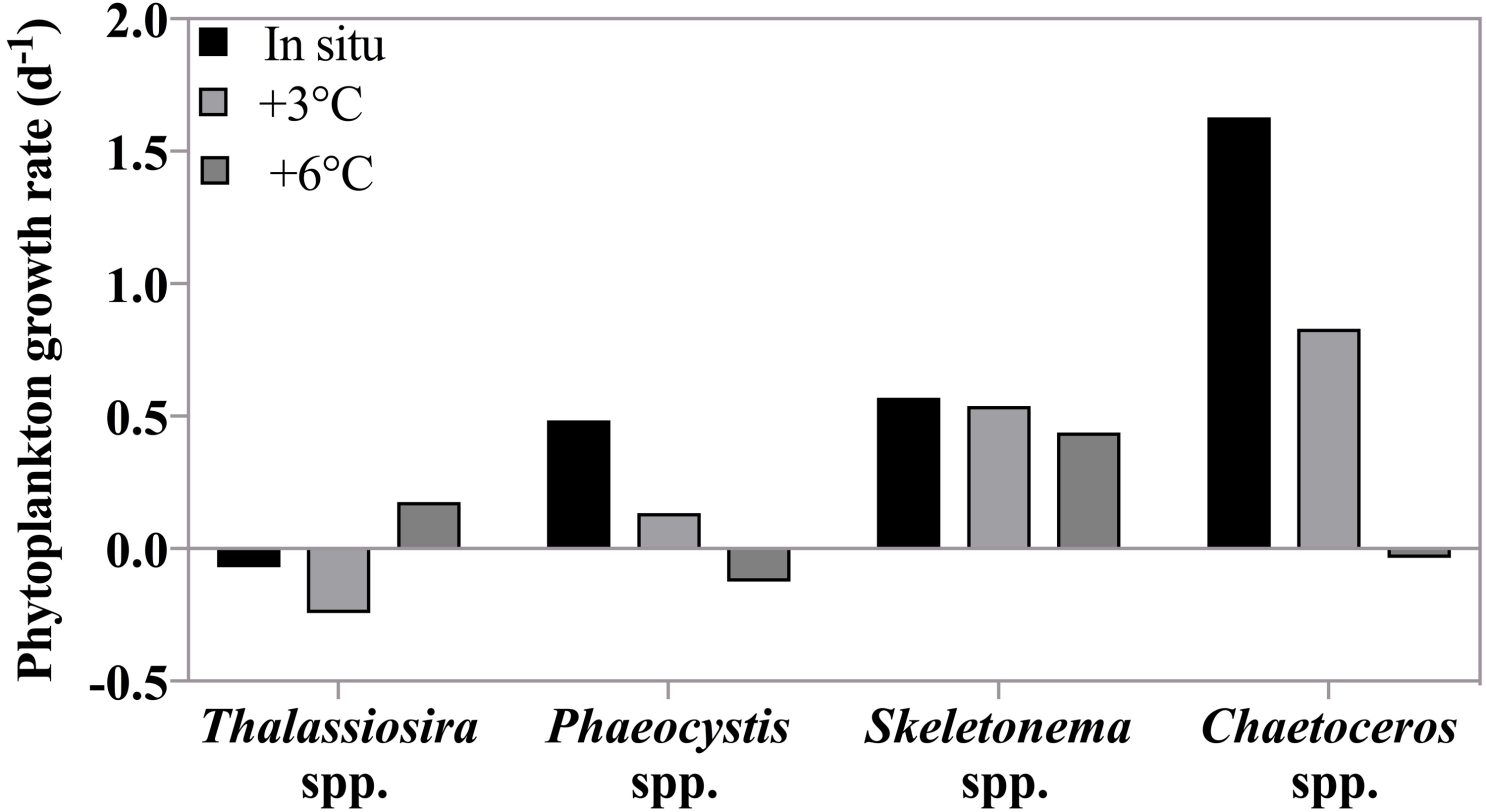
Phytoplankton growth rates as a function of incubation temperature. Growth rates (d^−1^) of four phytoplankton taxonomic groups at in situ, +3 °C and +6 °C on May 4th , when the highest grazing rates were measured. There was no significant effect of temperature on the growth rates of these species because both temperature dependent increases and decreases in growth rate were observed (see text).

Taxon-specific growth rates of phytoplankton showed high variability in response to increasing temperature indicating non-uniform responses to warming (Fig. 6). Overall, there was no significant response in phytoplankton group-specific growth rate to incubation temperature (2-way ANOVA, *p* = 0.27) as growth for some species increased with warming, while for others growth decreased. For instance, *Phaeocystis* spp. and *Chaetoceros* spp. were strongly affected by increasing temperature with much slower rates at +3 °C (0.13 and 0.83 d^−1^) compared to in situ, and negative growth at +6 °C (−0.12 and −0.03 d^−1^ respectively). On the other hand, *Thalassiosira* spp. grew more rapidly at +6°C (0.17 d^−1^) compared to the two lower temperatures where negative growth rates were observed. *Skeletonema* spp. growth rate remained the same at all temperatures. We note that *Skeletonema* spp. can include many cryptic species with broad and vast differences in temperature optima (Canesi & Rynearson, 2016) which might have masked a species-specific temperature response.

## Discussion

### Plankton growth and grazing at in situ, low temperatures

**Figure 7:**
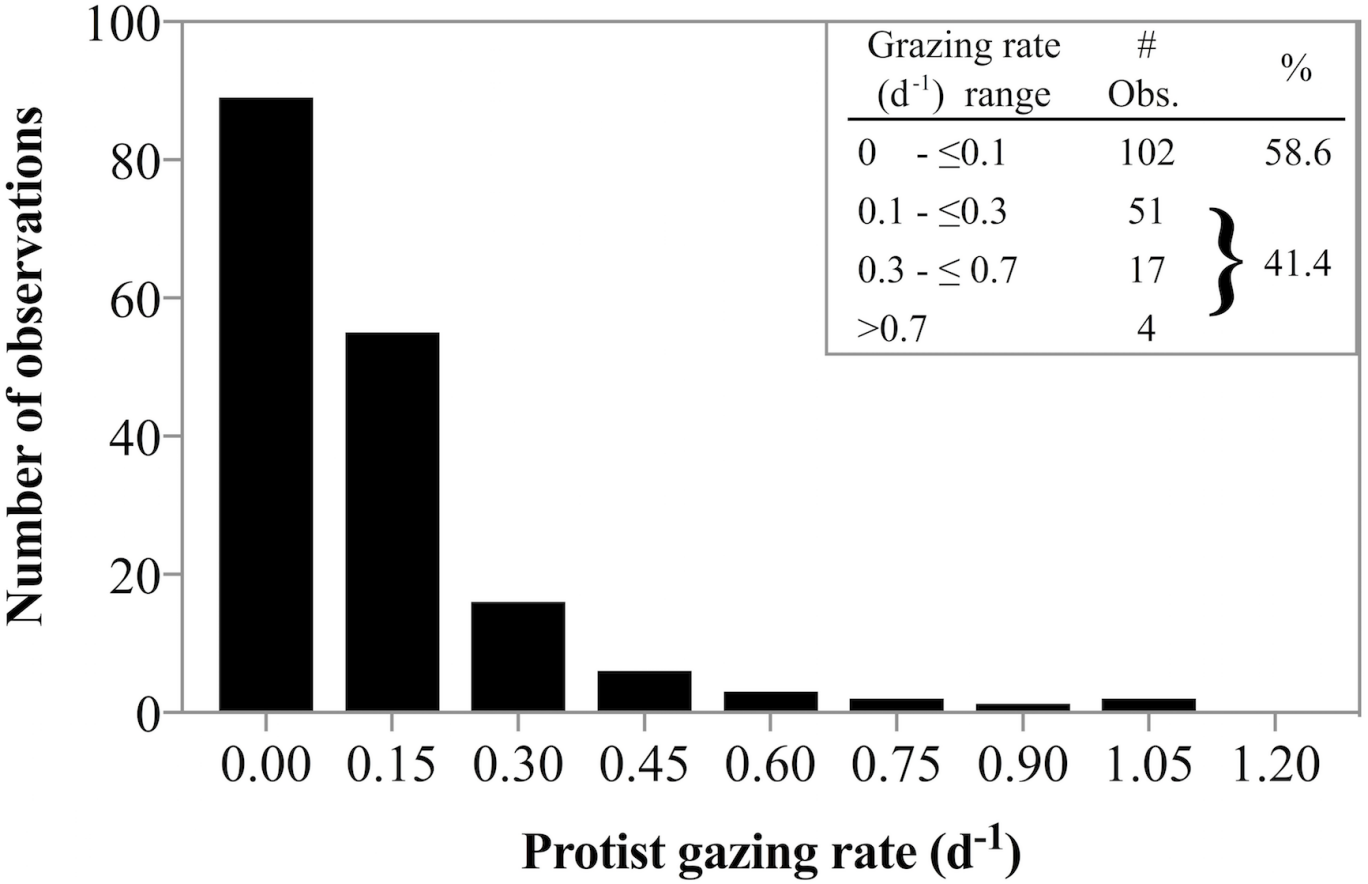
Frequency of low and high grazing rates made in diverse Arctic environments. Frequency of low and high grazing rates obtained from a literature survey of grazing rate measurements made in Arctic environments at temperatures ranging from −1.8°C to ≤10 °C. The data indicate near equal probability of detectable and undetectable herbivorous grazing. See text for references used to compile the data.

Estimating the relative rates of primary production and herbivorous grazing in sparsely sampled polar regions is important to gain a better understanding of the role of herbivorous protist grazing in high latitude food web dynamics and their potential response to seasonal and temperature shifts. In fact, Behrenfeld et al. (2017) argue that based on LIDAR observations, the annual cycle of polar plankton are well explained by lags between growth and loss processes. Our results are in good agreement with prior studies showing that at in situ, near-freezing temperatures, predator induced mortality for phytoplankton is often not detectable. The frequent absence of grazing documented in this study was punctuated by some measurements in which herbivores removed >100% of primary production. These switches between no-grazing to considerable grazing impact is well documented in the vast majority of measurements of herbivorous grazing in polar environments (Paranjape, 1987; Caron et al., 2000; Sherr, Sherr & Hartz, 2009; Calbet et al., 2011a; Calbet et al., 2011b; Sherr, Sherr & Ross, 2013; Franzè & Lavrentyev, 2017). In fact, combining our data with a literature survey of grazing rate data from available studies in arctic environments at temperatures from subfreezing (−1.8 °C) to ≤10 °C (Paranjape, 1987; Verity et al., 2002; Olson & Strom, 2002; Sherr, Sherr & Hartz, 2009; Calbet et al., 2011a; Calbet et al., 2011b; Sherr, Sherr & Ross, 2013; Franzè & Lavrentyev, 2017) reveals an interesting distribution of grazing rates. Of the 174 observation 59% suggest no measurable grazing, while 41% of rate estimates document some grazing impact (Fig. 7). However, the majority of the detectable grazing rates ranged between 0.1 and 0.3 d^−1^ (51 observation) resulting in an overall mean grazing rate measured in arctic waters of only 0.13 d^−1^. Such low rates could imply that herbivorous grazing cannot remove substantive amounts of phytoplankton production in polar regions. General averages yield low fractions of primary production removed. However, for those 3 dates when grazing rates were significantly greater than zero, ∼100% of primary production was consumed by herbivorous protists. It turns out, such high predator induced mortality rates are not uncommon in arctic environments (see Table 3 in Franzè & Lavrentyev, 2017). Similar dynamics of general lack of grazing punctuated by few but substantive grazing rates have been observed in sub-Arctic studies (Strom & Fredrickson, 2008) suggesting that to predict the population dynamics of predator and prey populations accurately, it may be more appropriate to parameterize protists grazing impact in high latitudes as an sometimes-on-frequently-off response, rather than a low average. The implications for the fate of phytoplankton production are that biomass fluctuations over time would be predicted to be more dynamic compared to a model with constant low losses.

Chl *a* concentrations recorded in this study were within the range of previously reported measurements from Disko Bay, ranging between a maximum of 24.1 µg L^−1^ in April 2008 (Dünweber et al., 2010) and a minimum of 5.9 µg L^−1^ in April 1998 (Levinsen et al., 2000). The increase in phytoplankton biomass observed is consistent with the rate measurements for both phytoplankton growth and predator induced mortality. The observed doubling in Chl *a* concentration shows that the phytoplankton community was not senescent or dying, as would be indicative of post-bloom conditions. In fact, to match the observed doubling of biomass in situ over a 9-day period, while ignoring other loss factors (e.g., viral lysis, sinking), requires an average daily growth rate of 0.08 d^−1^. This rate is well within the confidence interval of the average measured growth rate of 0.04 d^−1^. This agreement between measured rates and observed phytoplankton population dynamics suggests that phytoplankton growth and herbivorous protist grazing rates well capture the majority of the growth and loss processes driving biomass accumulation in the system.

In this study, herbivorous grazers grew at rates often higher than a doubling per day and, besides the temperature-dependent effects (see below), clearly thriving under experimental conditions. Species-specific growth rates for ciliates and dinoflagellates measured here were comparable to those from temperate regions (Nielsen & Kiørboe, 1994; Hansen, Bjornsen & Hansen, 1997) but contradict some previous low temperature studies, where maximum specific growth rates for ciliates and herbivorous dinoflagellates were measured to be ≤ 0.4 d^−1^ at temperatures of <2 °C (Bjornsen & Kuparinen, 1991; Hansen, Christiansen & Pedersen, 1996; Levinsen, Nielsen & Hansen, 1999; Møller, Nielsen & Richardson, 2006; Rose et al., 2013). Levinsen & Nielsen (2002) suggested that low temperatures explained low protistan growth rates in a prior study in Disko Bay. Nevertheless, our observations agree well with more recent observations from the Barents Sea where taxon specific growth rates of most ciliates and *Gymnodiniales* dinoflagellates reached their maximum growth rates at low temperatures (<5 °C) (Franzè & Lavrentyev, 2014). The observation made here that dinoflagellate growth rates exceeded those of ciliates are atypical (Strom & Morello, 1998), but not unusual for natural samples. Although ciliates have been observed to feed on large diatoms in sub-arctic waters (Aberle, Lengfellner & Sommer, 2007; Sherr, Sherr & Ross, 2013), diatoms are typically a more suitable prey type for large dinoflagellates than most ciliates (Jacobson & Anderson, 1986; Strom & Buskey, 1993; Naustvoll, 2000; Menden-Deuer et al., 2005; Sherr & Sherr, 2009). Thus, the dominance of diatoms as available prey might have favored dinoflagellate predators over ciliates. Additionally, it is not uncommon that incubations with whole plankton communities exhibit intraguild predation (Franzè & Modigh, 2013). Thecate dinoflagellates are well known predators of ciliates (Hansen, 1991; Stoecker & Evans, 1985; Smalley & Coats, 2002; Jeong et al., 2010) thus, the lower rates measured for ciliates might also reflect ciliate mortality due to dinoflagellate predation. Levinsen, Nielsen & Hansen (1999) also reported faster growth rates for dinoflagellates than ciliates in Disko Bay, possibly indicating that the conditions in this polar, coastal system favor more rapid dinoflagellate growth. Measurements of high species-specific growth rates at low temperatures imply that these herbivores could rapidly increase in abundance despite low ambient temperatures. Previous reports of almost instantaneous responses of herbivorous protists to spring bloom development in Disko Bay (Levinsen, Nielsen & Hansen, 2000) support this suggestion.

The observed variability in population growth rates could also contribute to the variability in measured grazing rates. Rose et al. (2013) found that although low temperatures constrained the growth rates of Antarctic ciliates, short-term ingestion rates were very high. A prior study by Sherr, Sherr & Hartz (2009) reported high grazing pressure at near freezing temperatures and maximal grazing rates. It appears that low temperature in and of itself does not limit the magnitude of measurable grazing rates.

The availability of suitable prey species, or lack thereof, is likely a key factor in generating the observed variability in measured herbivore growth rates (Irigoien, Flynn & Harris, 2005; Sherr & Sherr, 2009; Lawrence & Menden-Deuer, 2012). Indeed, we were able to document that *Phaeocystis* spp. had a significant negative impact on measured grazing rates. Calbet et al. (2011a) hypothesized that *Phaeocystis* spp. negatively impacted grazing rates and an adverse effect on grazing rates has previously been suggested by Gifford et al. (1995), who observed no measurable grazing by herbivorous protists during a *Phaeocystis* spp. bloom in the high-latitude North Atlantic. Caron et al. (2000) made similar observations in the Antarctic. In our study, availability of alternate prey such as diatoms was high. Thus prey availability was likely not a factor in causing low grazing rates. Instead, our results expand prior findings (Gifford et al., 1995) and show that there is not only a qualitative but rather a quantitative relationship between *Phaeocystis* abundance and decreased grazing pressure.

*Phaeocystis,* together with *Skeletonema*, which also contributed substantially to phytoplankton biomass, have been identified as major producers of secondary metabolites such as polyunsaturated aldehydes (PUA) (Hansen, Ernstsen & Eilertsen, 2004; Ribalet et al., 2007). Franzè et al. (2018) demonstrated that PUA primarily act as deterrent for herbivorous grazing. Thus, the strong negative effect of *Phaeocystis* abundance on grazing rate observed in this study might be the result of cytotoxic grazing deterrents of phytoplankton metabolites. These kinds of quantitative relationships may be useful to parameterize food web dynamics in models that make the important step of integrating the complexities of specific predator–prey species interactions.

Our results confirm and contribute to the mounting evidence that some factors commonly used to parameterize grazing rates are inappropriate predictors of exerted grazing pressure. There have been ample reports that bulk extracted Chl *a* concentrations and herbivorous protist abundance were not positively correlated with grazing rate (Strom et al., 2001; Olson & Strom, 2002; Verity et al., 2002; Sherr, Sherr & Hartz, 2009; Menden-Deuer & Fredrickson, 2010; Lawrence & Menden-Deuer, 2012). Various hypotheses have been advanced to explain the apparent lack of correlation between either Chl *a* concentration or grazer biomass and grazing pressure, including lack of suitable prey for present predator types during bloom initiation (Sherr & Sherr, 2009), predator selectivity due to chemical signaling (Olson & Strom, 2002), or simply inappropriate matching of predator–prey spectra (Irigoien, Flynn & Harris, 2005; Lawrence & Menden-Deuer, 2012). Such lack in correspondence between predator biomass and predation pressure is not universal. In a temperate estuary, Verity (1986) found a positive correlation between nano-phytoplankton abundance and ciliate grazing rates, likely due to the better matching of predator and prey types and size. However, for the large-diatom dominated communities studied here, and in the examples cited above, a size- or abundance-based driver of grazing rate was not identifiable and universal application of this parameterization in food web models may yield erroneous results.

### Biomass and composition

Phytoplankton abundance was vastly greater than herbivorous protist abundance during this spring bloom event, which differs from observations made at the same site in the summer, when the dominant size fraction of phytoplankton decreases from microplankton to nanoplankton (Nielsen & Hansen, 1999). Calbet et al. (2011a) also reported several occurrences of higher herbivorous than phototrophic carbon concentrations in the high Arctic. It is noteworthy that dinoflagellates were very abundant in our samples, comprising the majority of herbivorous protist biomass. This agrees well with previous studies of the plankton community composition in Disko Bay (Møller, Nielsen & Richardson, 2006; Madsen, Nielsen & Hansen, 2008), as well as with the abundances reported by Sherr, Sherr & Hartz (2009) for spring and summertime plankton in the Western Arctic Ocean. In our samples, *Gyrodinium spirale,* a species emerging as a particularly voracious consumer of phytoplankton, especially in high latitude marine microbial food webs (Sherr & Sherr, 2007), was a notable contributor to herbivorous biomass.

Copepods were deliberately eliminated from our experiments, as these larger predators are not adequately represented in liter-scale bottle incubations. Mounting evidence suggests that some copepod species preferentially feed upon herbivorous protists rather than phytoplankton (Calbet & Saiz, 2005 and references therein, Sherr & Sherr, 2009). Copepods’ selectivity towards heterotrophs has also been observed in the Arctic (Campbell et al., 2009; Campbell et al., 2015) and herbivorous protists have been shown to be an important prey source for copepods in Disko Bay (Riisgaard et al., 2014). Moreover, two field studies reported feeding preference on herbivorous prey, rather than phytoplankton even when diatom prey were abundant (Levinsen et al., 2000; Campbell et al., 2009). Our approach to exclude copepods from incubations could result in an overestimate of herbivorous grazing rates, when copepods are abundant and preferentially feed upon herbivorous protists. Nonetheless, on the dates when significant grazing was observed, the percent primary production consumed by herbivorous protists (minimum 25%) was on par with the maximum and greater than the average grazing impact measured for copepods in comparative studies (Campbell et al., 2009). Moreover, copepod predation impact varies with time. For instance, *Calanus* spp. feeding rates are known to decrease during the summer (Levinsen et al., 2000) due to lower predator abundance (Madsen, Nielsen & Hansen, 2001; Swalethorp et al., 2011). Studies utilizing natural plankton assemblages and direct estimates of both copepod and herbivorous protist grazing impact typically conclude that copepods consume generally less than 25% primary production. Thus, in comparison, herbivorous protist grazing appears to remove a greater fraction of primary production than grazing by copepods.

### Temperature effects

Our measurements of un-acclimated growth and grazing rates indicate that rapid changes in temperature, which are expected as climate change induces greater environmental variability, affect phototrophs and heterotrophs differently. The resulting decrease in the fraction of primary production consumed—high at low temperatures, low at high temperatures—would represent an unexpected qualitative and quantitative shift in trophic transfer vs. export production rates.

**Figure 8:**
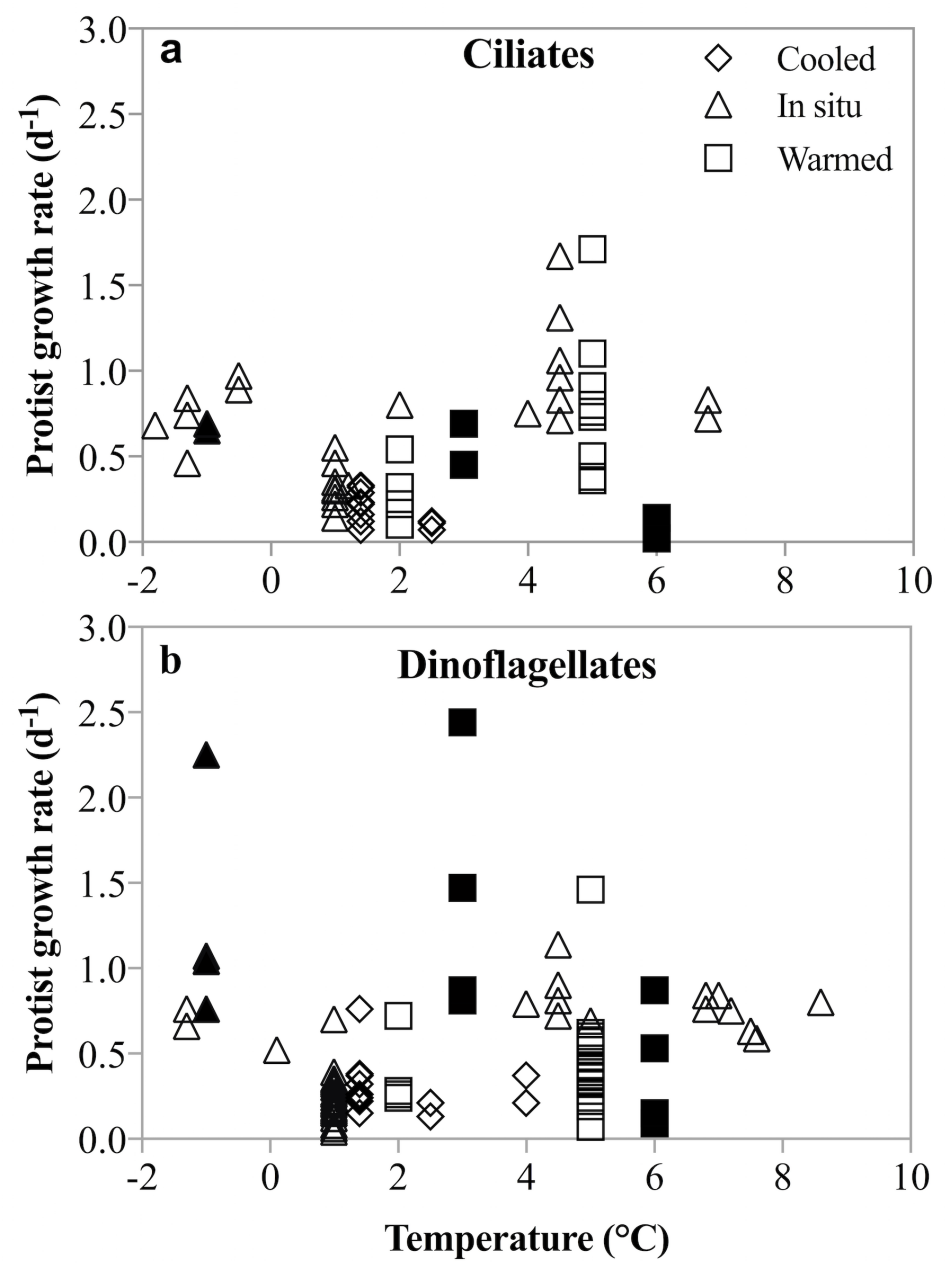
Un-acclimated ciliate and dinoflagellate growth rates in response to temperature shifts. Literature compilation of ciliate (A) and dinoflagellate (B) growth rates at temperatures between −1.8 and 8.6°(C). The rate measurements were made on samples incubated at in situ (triangles), un-acclimated warmer (squares) and un-acclimated colder (diamonds) temperatures. Filled symbols are rates measured in this study; open symbols are from: Levinsen, Nielsen & Hansen (1999), Hansen & Jensen (2000), Seuthe, Iversen & Narcy (2010), Møller, Nielsen & Richardson (2006), Franzè & Lavrentyev (2014), Bjornsen & Kuparinen (1991).

No acclimation of the mixed plankton communities was undertaken, so the temperature-dependent changes in growth and grazing rates mimic most closely a short-term disturbance to the system. The differential response of phytoplankton and herbivorous protists growth and grazing rates may be an expression of a short-term shock response to this rapid temperature change. However, a number of studies and coincidental observations have demonstrated that lack of acclimation does not result in unusual or depressed rate measurements (Levinsen, Nielsen & Hansen, 1999; Hansen & Jensen, 2000; Seuthe, Iversen & Narcy, 2010; Møller, Nielsen & Richardson, 2006; Franzè & Lavrentyev, 2014; Bjornsen & Kuparinen, 1991). In these studies, dinoflagellate and ciliate growth rates in un-acclimated incubations that were either cooled (Δ −0.5 to −3.6 °C) or warmed (Δ 1.5 to 7 °C) were comparable to those measured at in situ temperature. An anticipated depression of rates due to the presumable shock of rapid temperature shifts was not observed (Fig. 8). Similarly, herbivorous protists exposed to an unexpected increase in temperature of 4.5 °C over 24 h, did not show either a precipitous decline or significantly faster growth compared to growth at in situ temperatures (Franzè & Lavrentyev, 2014). Finally, species-specific growth rates of herbivorous protists measured here are comparable to temperate rates, despite the lack of acclimation (Hansen, Bjornsen & Hansen, 1997; Rose & Caron, 2007). Thus, notwithstanding acclimation caveats, our results contradict the suggestion that low temperatures constrain the intrinsic growth and grazing rates of herbivorous protists, and that increasing temperatures result in more rapid increases in herbivorous than phototrophic growth rates. The implications for modeling plankton population dynamics on a warming planet are substantial.

A positive correlation between temperature and phytoplankton growth rates has been well established by Eppley (1972), and ample empirical evidence supports it (Cossins & Bowler, 1987; Bissinger et al., 2008; Kremer, Thomas & Litchman, 2017). The expectation of increased phytoplankton growth rates at higher temperatures have led to predictions of significant changes in plankton population dynamics and community composition as a function of climate change. These include an earlier onset of spring bloom events (Weyhenmeyer, Blenckner & Pettersson, 1999; Stenseth & Mysterud, 2002; Stenseth et al., 2002), and a significant decrease in primary production due to the greater light availability earlier in the year (Polovina, Howell & Abecassis, 2008; Doney et al., 2009). Earlier light availability due to decreasing sea ice cover (Nghiem et al., 2007) could lead to a wholesale shift in phytoplankton species composition, and thus lead to an earlier depletion of nutrients and a lower phytoplankton biomass in ‘spring’. Future decreases in primary production are expected to be most pronounced at high latitudes (Gregg et al., 2003). Empirical observations using satellite data confirm large-scale, long-term decreases in primary production coincident with warming trends (Behrenfeld et al., 2006). In contrast, some studies forecast future increases in primary production due to increased wind-induced upwelling of nutrient rich waters (Arrigo, Van Dijken & Bushinsky, 2008). Temperate species may also shift pole-ward (Barton et al., 2016). However, important unknowns, including the effects of rapidly changing seawater carbonate chemistry (Orr et al., 2009) on organismal physiology, and food web structure and function (reviewed in Caron & Hutchins, 2012), make predictions of future plankton production rates difficult and highly uncertain.

**Figure 9:**
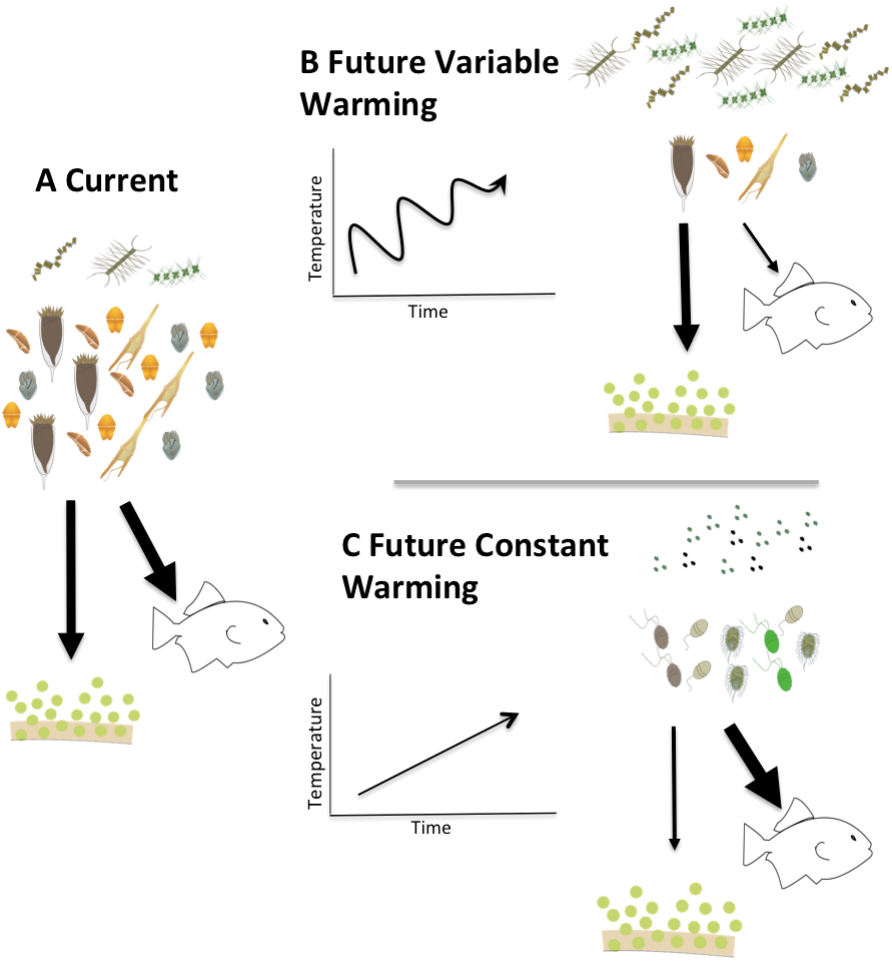
Conceptual model of the fates of primary production in fluctuating vs. constantly increasing warming conditions. Conceptual model describing two possible future scenarios of temperature induced shifts in Arctic planktonic food webs. Steady temperature increase (C) could elicit a microbial community dominated by picoplankton similar to more temperate systems while warming events alternating with cooling (B) could exaggerate the mismatch between production and consumption and promote a microplankton dominated food web with a relatively larger amount of export production.

Clearly, much needs to be done to unravel the mechanisms that drive the physiological responses of plankton to temperature changes, and particularly potential differences in response between trophic modes. Our observations suggest that rapid, short-term changes in temperature might have different ramifications than constant increases in temperature. Summarizing these implications in a conceptual model (Fig. 9) illustrates that a constant increase in temperature (Fig. 9C) would provide time for both autotrophs and heterotrophs to acclimate to warmed conditions promoting a transition to picoplankton and to a microbial loop dominated systems. The changes in the microbial compartment would likely cascade throughout the entire food web favoring the transfer of carbon to higher trophic levels, with relatively smaller losses due to sedimentation, sensu Moran et al. (2010). On the other hand, in a system exposed to rapid fluctuations of warming and cooling (Fig. 9B), acclimation would not be possible and the mismatch between autotrophic growth and herbivorous grazing rates we document here could exaggerate the asynchrony in increased production and decreased herbivory, yielding a higher degree of export production and lower carbon availability for transfers through the pelagic food web. Measurements of the temperature dependence of herbivorous growth and grazing rates are urgently needed to anticipate how matter and energy will flow through a warming polar food web.

## Conclusion

Overall, we found that microplankton growth and grazing rates could occasionally be comparable to those measured in coastal temperate regions, and thus phytoplankton growth and herbivorous grazing are important factors in the transfer of matter and energy in Arctic food webs. The widely reported high variability in herbivorous protist grazing rates that we also observed implies rapid fluctuations in phytoplankton biomass are to be expected. We did find *Phaeocystis* abundance to be a quantitative and biotic driver of low grazing rates, but found no evidence for an absolute and inherent suppression of herbivorous protist growth and grazing rates due to low temperature alone. Herbivorous protists physiology is evidently much more susceptible to short term temperature shock than the apparently more robust phytoplankton. This differential response implies that temperature increases do not instantaneously increase growth and grazing rates of herbivorous protists. Therefore, an upward “correction” of herbivorous growth and grazing rates using static correction factors (e.g., Q10) and ignoring acclimation potential, may overestimate herbivorous feeding and growth rates, and thus the fate of primary production, in high latitude planktonic food webs.

## Additional Information and Declarations

### Data Availability

The following information was supplied regarding data availability: Environmental and Biological Observations (data_1): https://www.bco-dmo.org/dataset/739708
Carbon Biomass (data_2): https://www.bco-dmo.org/dataset/739750
Growth Rates (data_3): https://www.bco-dmo.org/dataset/739806.
